# Ferroelasticity and domain physics in two-dimensional transition metal dichalcogenide monolayers

**DOI:** 10.1038/ncomms10843

**Published:** 2016-02-24

**Authors:** Wenbin Li, Ju Li

**Affiliations:** 1 Research Laboratory of Electronics, Massachusetts Institute of Technology, Cambridge, Massachusetts 02139, USA; 2 Department of Nuclear Science and Engineering and Department of Materials Science and Engineering, Massachusetts Institute of Technology, Cambridge, Massachusetts 02139, USA

## Abstract

Monolayers of transition metal dichalcogenides can exist in several structural polymorphs, including 2H, 1T and 1T′. The low-symmetry 1T′ phase has three orientation variants, resulting from the three equivalent directions of Peierls distortion in the parental 1T phase. Using first-principles calculations, we predict that mechanical strain can switch the relative thermodynamic stability between the orientation variants of the 1T′ phase. We find that such strain-induced variant switching only requires a few percent elastic strain, which is eminently achievable experimentally with transition metal dichalcogenide monolayers. Calculations indicate that the transformation barrier associated with such variant switching is small (<0.2 eV per chemical formula unit), suggesting that strain-induced variant switching can happen under laboratory conditions. Monolayers of transition metal dichalcogenides with 1T′ structure therefore have the potential to be ferroelastic and shape memory materials with interesting domain physics.

## Abstract (web summary)

The atoms in two-dimensional transition-metal dichalcogenides can arrange into a number of different structures, or polymorphs. Here, the authors use first-principles calculations to show that one such polymorph, 1T', can exhibit a large mechanical response to external applied strain.

## Graphical abstract

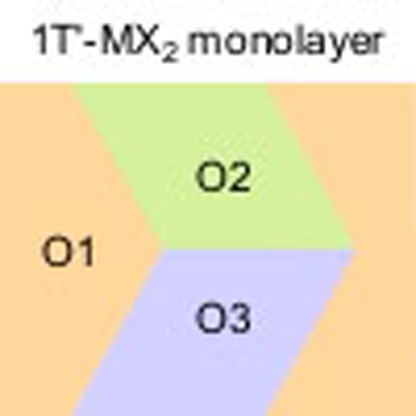

The discovery of two-dimensional (2D) atomic crystals^1^ has fuelled intensive research efforts on this new class of materials, revealing fundamentally new physics and properties^2^,^3^,^4^,^5^,^6^ that could be essential for next-generation nanoscale devices. Monolayers of group VI transition metal dichalcogenides (TMDs) with chemical formula MX_2_, where M is Mo or W and X stands for S, Se or Te, have in particular attracted much recent attention due to their semiconducting, optical and valleytronic properties^4^,^7^,^8^,^9^. Owning to their atomic thickness, the TMD monolayers have extraordinary mechanical flexibility and strength, capable of sustaining up to 10% of elastic strain before failure^10^,^11^, which enables significant dynamical tuning of their properties by strain engineering^12^ and makes them attractive for application in ultrathin flexible electronics^13^,^14^,^15^.

MX_2_ monolayers can exist in several polytypic structures, including 2H, 1T and 1T′^16^,^17^,^18^. In the semiconducting 2H phase, the atomic stacking sequence within a single XMX monolayer is Bernal (ABA) and the M–X coordination is trigonal prismatic. In contrast, in the 1T phase, the XMX stacking sequence is rhombohedral (ABC), and the M and X atoms form octahedral coordination. The 1T phase is metallic, but was found to be unstable to Peierls distortion^19^,^20^, where two adjacent lines of metal atoms along the highest symmetry directions can dimerize and form parallel chains of M atoms. This leads to the formation of 1T′ phase^17^,^18^, in which the octahedral coordination between M and X atoms becomes distorted, and the symmetry of the crystal structure is reduced. While the thermodynamically stable phase of most group VI MX_2_ monolayers under ambient conditions is 2H, the ground-state phase of WTe_2_ has 1T′ structure^16^,^21^. For other MX_2_ monolayers, the 1T′ phase is usually metastable, but large transition barriers of order 1 eV per formula unit exist between 1T′ and 2H (ref. 22), suggesting that the 1T′ phase can be stabilized under appropriate thermal or chemical conditions. In particular, the energetic difference between the 2H and 1T′ phase of MoTe_2_ is rather small^19^, suggesting that the 1T′ phase can be stabilized relatively easily. Indeed, single crystals and few-layer films of MoTe_2_ in 1T′ phase have been synthesized on a large scale recently^20^,^23^,^24^. It has also been theoretically proposed that the 2H to 1T′ transition in MoTe_2_ monolayers can be induced by experimentally accessible tensile strain^19^.

The low-symmetry 1T′ phase of TMD monolayers harbours extraordinary properties that have only started to be revealed, which, for example, includes enhanced catalytic activities^25^, large, non-saturating magnetoresistence^21^ and quantum spin Hall effect^22^.

A ferroelastic material is defined by the existence of two or more equally stable orientation variants, which can be switched from one variant to another without diffusion by the application of external stress^26^,^27^. A ferroelastic phase usually forms through a structural phase transition (or a hypothetical one) that reduces the symmetry of a prototype phase. The low-symmetry ferroelastic phase possesses several orientation states (domain variants) with different spontaneous strain^28^, that is, the distortion of the unit cell relative to that in the prototype phase. The difference in spontaneous strain between different variants enables external stress to couple energetically with the strain state of the system and drive orientation switch, analogous to the switching of spontaneous polarization by external electric field in a ferroelectric material. In a ferroelastic crystal, domains of different orientations can coexist and form twin boundaries. On activation by appropriate external stress, those twin boundaries can move in a glissile fashion, resulting in the growth of one orientation state at the expense of another, as well as hysteretic stress–strain response^27^.

In this article, we focus on the possibility of ferroelastic behaviours in 1T′–MX_2_ monolayers. A notable feature associated with the 1T′ phase that has hitherto been overlooked is that it has three distinct orientation variants, resulting from the three equivalent directions of structural distortion in the parental 1T phase. Our density functional theory (DFT) calculations indicate that ferroelastic switching can occur between the different orientation variants of the 1T′ phase with a few percent of elastic strain, which is experimentally achievable for MX_2_ monolayers.

## Results

### Crystal structures and transformation strains

**Figure 1:**
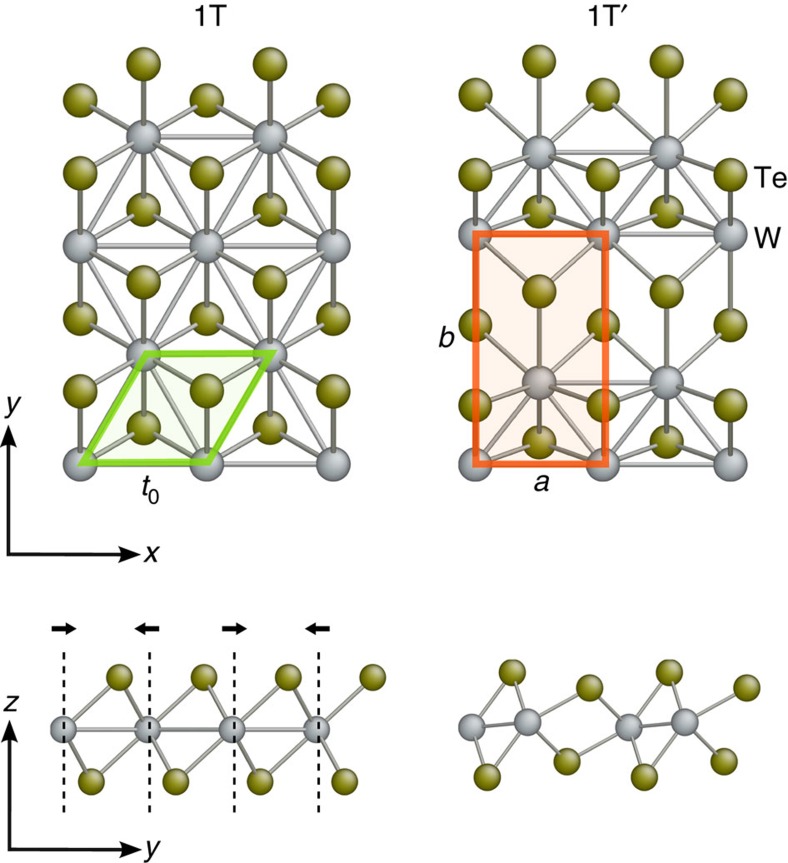
Atomistic structure of of 1T–WTe_2_ and 1T′–WTe_2_ monolayers. The 2D primitive cells of 1T and 1T′ are highlighted in green and red, respectively. The primitive cell of 1T′ corresponds to the 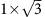 supercell of 1T. The 1T′ phase can be derived via the structural distortion of the 1T phase, which is schematically illustrated in the side views. These features are generic to all other group VI MX_2_ monolayers.

**Figure 2:**
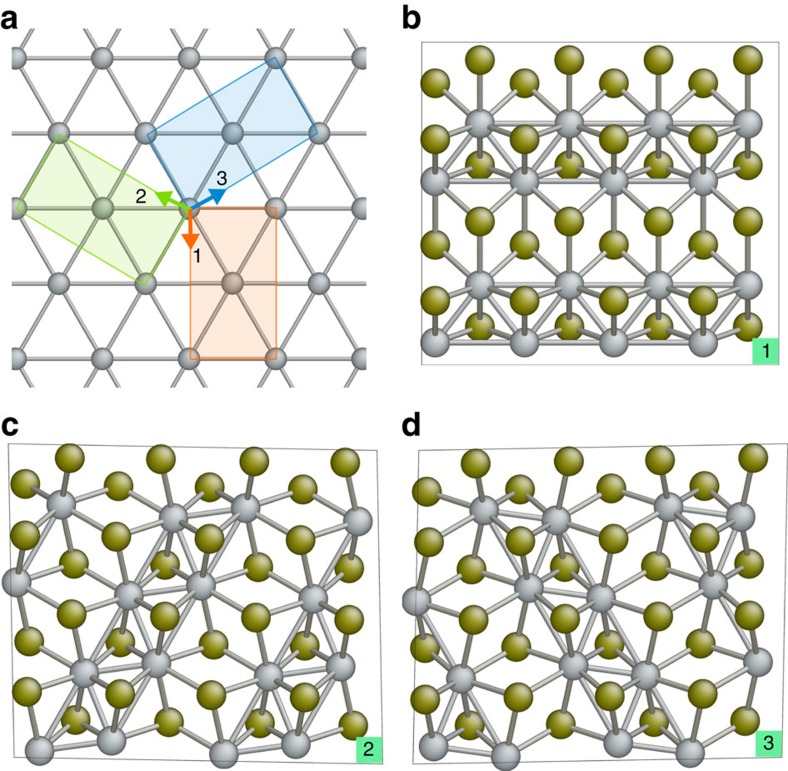
Three orientation variants of 1T′–MX_2_ monolayers. (**a**) The three symmetry-equivalent directions of structural distortion in the 1T structure are indicated by arrows and numerical labels on the triangular lattice formed by M atoms. The corresponding primitive cells of the 1T′ phase after structural distortion are represented as shaded rectangles. (**b**–**d**) Relaxed atomistic structure of the 1T′ phase after structural distortion along the three different directions, which are referred to as orientation variants O1, O2 and O3, respectively.

We use WTe_2_ monolayers as a representative of 1T′–MX_2_ to illustrate the possibility of 2D ferroelasticity. Figure 1 shows the atomistic structures of 1T–WTe_2_ and 1T′–WTe_2_ monolayers. In the 1T phase, the W atoms arrange in 2D triangular lattice, which is sandwiched between two Te atomic layers. The 2D primitive cell of the 1T phase is a 120° rhombus with side length *t*_0_. Due to Fermi surface nesting induced Peierls distortion^20^, adjacent parallel lines of W atoms along the high-symmetry [100], [010] or [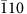] directions in the 1T structure can spontaneously dimerize and result in the formation of 1T′ phase, with distorted octahedral coordination. The 2D primitive cell of the 1T′ phase is a rectangle with dimensions *a* × *b*, which corresponds to the 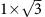 supercell of the 1T phase. Because of the 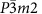 space group symmetry of the 1T phase, there are three symmetry-equivalent directions of structural distortion in the 1T phase. These directions are labelled on Fig. 2a as direction 1, 2 and 3 on the 2D triangular lattice formed by W atoms. The atomistic structures of the three orientation variants formed by structural distortion in the 1T phase along the three directions are shown in Fig. 2b–d. Hereafter, we refer to the three orientation states as the O1, O2 and O3 variant, respectively.

The spontaneous transformation strains associated with the 1T to 1T′ transformation can be compared between the three variants based on the 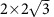 supercell of the prototype 1T phase. All the three orientation variants of the 1T′ phase, namely, O1, O2 and O3, can be derived through the Peierls distortion of this supercell and the atoms within the supercell along the corresponding orientation direction. Namely, the 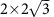 supercell of 1T can transform to become the supercells of all three variants of the 1T′ phase. In Cartesian coordinates, the 2D basis vectors **h**_1_ and **h**_2_ of the 1T supercell can be written as 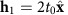, 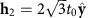, where 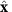 and 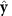 are the unit vectors along the *x* and *y* directions labelled on Fig. 1. A supercell matrix **H**_0_={**h**_1_,**h**_2_} can be constructed, where **h**_1_ and **h**_2_ are treated as column vectors, that is,


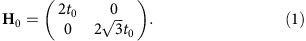


After transforming to the 1T′ phase, the distorted supercell matrix corresponding to the O1, O2 and O3 variants will be denoted by **H**_1_, **H**_2_ and **H**_3_, respectively. These new supercell matrices can be related to the original supercell matrix by transformation matrices **J**_*i*_, which map the undistorted supercell to the distorted supercells. Namely, **H**_*i*_=**J**_*i*_**H**_0_, where the subscript *i* stands for the *i*-th orientation variant. The transformation strain matrices **η**_*i*_ associated with different variants can then be calculated from **J**_*i*_ based on the definition of Green-Lagrange strain tensor:


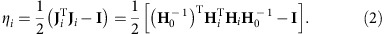


Here, the superscripts −1 and T denote matrix inversion and transposition, respectively. **I** is a 2 × 2 identity matrix. The 2D transformation strain tensor **η**_*i*_ has the following symmetric form:


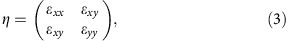


where *ɛ*_*xx*_ and *ɛ*_*yy*_ are the tensile/compressive strain along *x* or *y* direction, and *ɛ*_*xy*_ is the shear strain component.

We have employed DFT calculations to obtain the equilibrium supercell vectors and the relaxed atomic coordinates of the O1, O2 and O3 variants, resulting from the distortion of 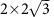 supercell in the 1T prototype phase. The supercell matrices **H**_*i*_ for different MX_2_ monolayers are tabulated in Supplementary Table 1. From the supercell matrices, the spontaneous transformation strain tensors **η**_*i*_ can be evaluated, which are listed in Supplementary Table 2. For WTe_2_ monolayers, the transformation strain matrices form 1T to 1T′ are


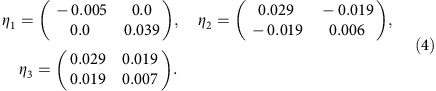


The difference in transformation strain between the three variants of 1T′ suggests that one may switch the relative thermodynamic stability between different variants by applying suitable external mechanical stress. Since the equilibrium structure of WTe_2_ is 1T′, it is informative to directly compare the distorted supercell of the three variants by computing the relative supercell strain associated with the transformation from one variant to another. This can be carried out again using the supercells of the three variants derived from the common 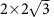 supercell in the prototype 1T phase. The reference configuration for computing the supercell strain is now chosen to be the O1 variant of 1T′ phase, and we use 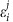 to denote the transformation strain tensor from variant *i* to *j*. Calculations based on the same definition of strain tensor as in equation (2) give the transformation strain associated with O1–O2 and O1–O3 switching to be


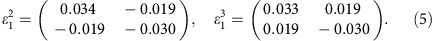


It then follows that, starting with the O1 variant of 1T′ in a strain-free state, after imposing an external strain of magnitude 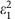 on the monolayer, the system would be in a thermodynamically more favourable state by transforming to the O2 variant, since both O1 and O2 belong to the same 1T′ structure, but O1 will have higher strain energy than O2. The same argument applies to any other two variants. Hence, the relative energetic stability between the different orientation variants of 1T′ phase can be controlled by external stress or strain.

### Variant energetics under biaxial and shear strain

To study in detail the relative thermodynamic stability of different variants when external mechanical deformation is imposed on a 1T′–MX_2_ monolayer, we have used DFT to calculate the potential energy surfaces of the three variants of 1T′ as a function of 2D supercell dimensions. We first investigate the possibility of mechanically switching the O1 variant to O2 or O3 variant by applying biaxial strain to the system, again using WTe_2_ monolayer as an example. The strain-free 2 × 2 supercell of the O1 variant, derived from the distortion of the aforementioned 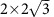 supercell in the parental 1T phase, is chosen to be the reference system. The 2 × 2 supercell of the O1 variant has dimensions 2*a* × 2*b* within the *x*–*y* plane of 2D monolayer. We adjust the dimensions of the supercell along *x* and *y* directions independently, with the values of *a* and *b* range from −10 to 10% of engineering strain at an equal step of 2%. At each pair of (*a*,*b*), the atomic coordinates within the supercell are relaxed. We also compute the energies of O2 and O3 variants when their supercell dimensions are fixed to be the same as O1. The energies *U* of all three variants are computed on a 11 × 11 grid in the (*a*,*b*) space, giving a total number of 121 data points distributed evenly around the equilibrium lattice constants of the O1 variant. Smooth potential energy surfaces are then constructed by approximating the intermediate values of *U*(*a*,*b*) using 2D spline interpolation, which allows us to directly compare the relative energetic stability of the O1, O2 and O3 variants in the full (*a*,*b*) space. In addition, the *U*(*a*,*b*) for the 2H phase is computed for comparison, as a previous study indicates that strain-induced phase transformation between the 2H and 1T′ phases can happen in MX_2_ monolayers^19^.

**Figure 3:**
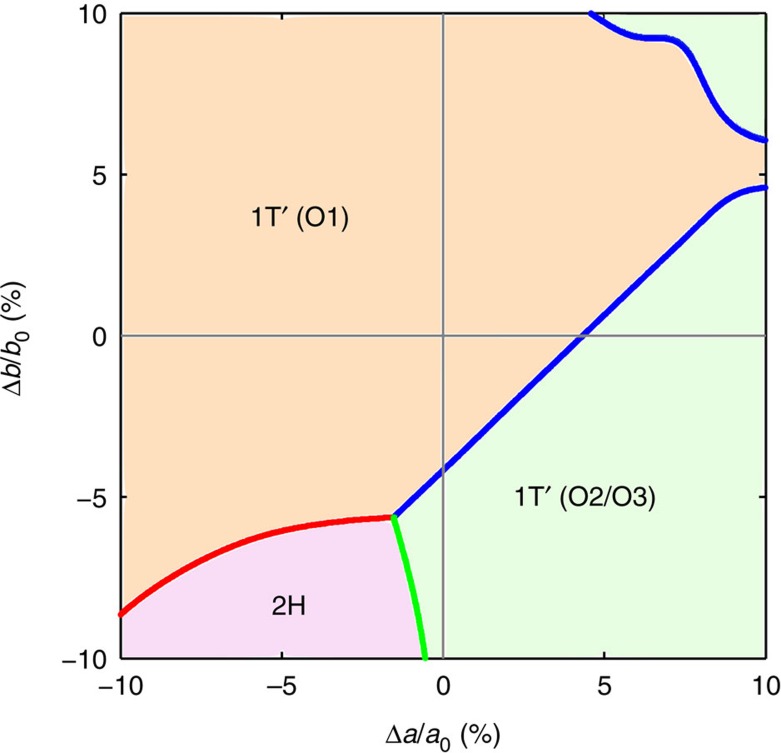
Intersection contours of the energy surfaces between the different orientation variants of 1T′–MoTe_2_ and between 2H and 1T′. The lattice constants *a* and *b*, corresponding to the dimensions of the rectangular primitive cell of the O1 variant in the 1T′ phase, are represented as percent engineering strain with respect to the equilibrium lattice constants *a*_0_ and *b*_0_. The regions of lower-energy phase/variant are labelled and shaded in different colours.

**Figure 4:**
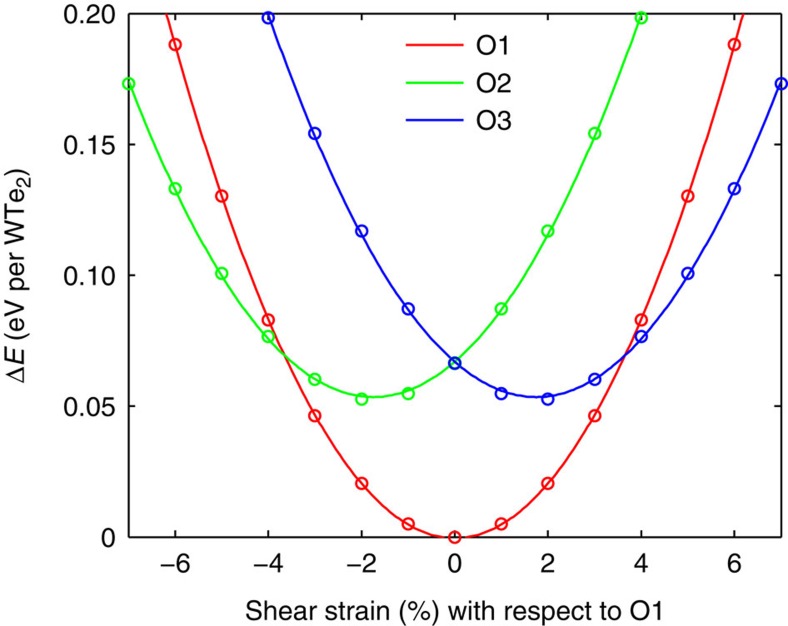
Potential energies of the three variants of 1T′–WTe_2_ monolayers as a function of shear strain with respect to the equilibrium supercell of the O1 variant. The O2 or O3 variant becomes energetically more favourable when negative- or positive-shear strain of a few percent is imposed on the O1 variant.

After obtaining the potential energy surfaces for all the three variants of 1T′ as well as the 2H phase, the lowest-energy variants/phases in the (*a*,*b*) space are determined. The result is shown in Fig. 3, where we label the lowest-energy variant/phase in each region of phase space and plot the intersection boundaries between two neighbouring variants/phases. An important feature of Fig. 3 is that the potential energy surfaces of O1 and O2/O3 variants intersect at a few percent of biaxial supercell strain, which is experimentally achievable in MX_2_ monolayers^10^,^11^. The O2 and O3 variants are grouped together in Fig. 3 because their potential energies in the (*a*,*b*) space are essentially the same. This can be rationalized by the fact that the supercells of both variants can be derived from the distortion of the 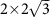 supercell in the 1T phase, and their distortion directions are related by mirror symmetry along the *y* direction in the 1T phase, as can be seen from Fig. 2. Since biaxial strain does not break the mirror symmetry of 1T phase along the *y* axis, the O2 and O3 variants are still mirror images of each other and have the same energy. We however expect that shear strain, which breaks the mirror symmetry, can distinguish the energies of all the three variants of 1T′. Indeed, Fig. 4 shows that, when shear strain *ɛ*_*xy*_ of magnitude >3.5% is imposed on the O1 variant, O3 becomes the lowest-energy variant within the strained supercell. If the sign of *ɛ*_*xy*_ is reversed, then the O2 variant has lower energy than both O1 and O3.

Figure 3 indicates that the 2H phase of WTe_2_ monolayer only takes a small region in the (*a*,*b*) space as the lowest-energy phase. This result is different from the study by Duerloo *et al*.^19^ of strain-induced phase transformation between the 2H and 1T′ phases of MX_2_ monolayers, as the authors did not take into account the existence of orientation variant degrees of freedom in the 1T′ phase.

Figure 3 also shows that the fastest route to switching the energetic order between O1 and O2/O3 in the (*a*,*b*) space is by applying tensile strain along the *a* axis of the O1 variant, which is the direction of dimerized metal-atom chains, while simultaneously applying compressive strain along the *b* axis. It is however known that 2D MX_2_ monolayers usually cannot sustain large compressive strain due to compression-induced buckling response and formation of ripplocations^29^. On the contrary, experiments have demonstrated that 2H–MX_2_ monolayers can withstand tensile elastic strain as large as 10% before mechanical failure^10^,^11^. Hence, it may be experimentally more convenient to realize variant switching in 1T′–WTe_2_ by uniaxially stretching it along the *a* axis, which is the direction of dimerized tungsten atoms. This axis can be identified by mechanical cleavage or by the anisotropic response to external fields that is expected for the low-symmetry 1T′ structure^21^.

In Supplementary Fig. 1, we have also computed the intersection contours of the potential energy surfaces between the O1 and O2/O3 variants for other 1T′–MX_2_ monolayers, including MoS_2_, MoSe_2_, MoTe_2_, WS_2_ and WSe_2_. The results are very similar to WTe_2_, indicating that strain-induced switching of thermodynamic stability between different orientation variants is generic to MX_2_ monolayers with 1T′ structure.

### Variant energetics under uniaxial tension

**Figure 5:**
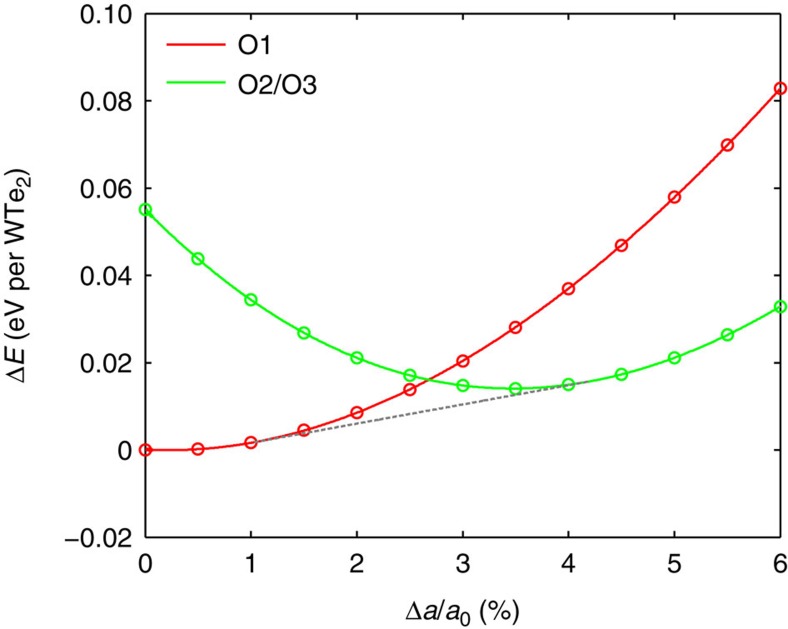
Potential energy curves of the different variants of 1T′–WTe_2_ uniaxially strained along the *a* axis. The dashed line is the common tangent between the two curves. The uniaxial strain is calculated with respect to the equilibrium supercell dimension of the O1 variant along the *x* direction.

We emphasize that the strain at which the potential energy surfaces of different variants intersects is not the same as the strain at which variant switching becomes thermodynamically favourable. The system can minimize its free energy by choosing a state where different variants (or phases) coexist, akin to the two-phase region in chemical-composition phase diagrams. Under constant temperature and fixed external strain (supercell dimensions), the thermodynamic potential that determines the relative variant/phase stability is the Helmholtz free energy *F*=*E*—*TS*, where *E* is internal energy that includes both potential energy *U* and kinetic energy, *T* is temperature and *S* is entropy. Because all the three variants O1, O2 and O3 belong to the same 1T′ structure, and because the entropy of solids (mainly vibrational) is relatively insensitive to small deformation, we can use the potential energy *U* of different variants, computed by DFT at zero temperature, to compare the free energies of different variants at ambient conditions. In Fig. 5, we plot the potential energy curve of the O1 variant of 1T′–WTe_2_ when it is uniaxially stretched along the *a* axis. Consistent with typical experimental set-ups for uniaxial deformation, the stress of the supercell along the *b* axis is relaxed to zero. This corresponds to free boundary, or zero stress (*σ*_*y*_=0) condition along the *b* axis. In Fig. 5, we also plot the potential energy of the O2/O3 variant in a rectangular supercell with the same dimension along the *a* axis and the same boundary condition along the *b* axis. A common tangent can be constructed between the energy curves of O1 and O2/O3, which intersects the two curves at uniaxial strains equal to 1% and 4%, respectively. Between these two values, the system can lower its energy by existing in a state where both O1 and O2/O3 variants coexist. This indicates that the formation of O2/O3 variants becomes thermodynamically favourable when the uniaxial strain along the *a* axis of O1 is as low as 1%.

### Kinetic aspects of variant switching

**Figure 6:**
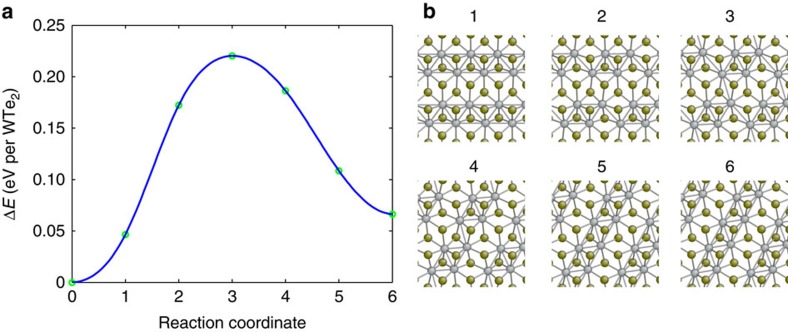
NEB calculation of transformation barrier and the pathway for orientation switching. The initial configuration of the NEB calculation is the O1 variant of 1T′–WTe_2_ monolayers at ground state (zero external stress), while the final state is the strained O2 variant with the same supercell dimensions as those of the O1 variant. (**a**) Change of the system energy per chemical formula unit as a function of reaction coordinate. (**b**) The corresponding atomistic structure of the system along the reaction coordinate. Four different supercells were used to carry out the NEB calculations: 2 × 2, 2 × 4, 4 × 2 and 4 × 4 supercell of the 1T′ phase, which all give identical results.

Up to now, we have only considered the thermodynamic aspects of variant switching in the 1T′–MX_2_ monolayers. Our results suggest that it becomes thermodynamically favourable for the O1 variant of 1T′–WTe_2_ monolayers to switch to the other two variants when applying uniaxial strain around 1% along the direction of dimerized tungsten atom chains. However, if the kinetic barrier associated with variant switching is too high, such variant switching may not occur under normal experimental conditions and timescale, and the materials would still not be ferroelastic. We have therefore computed the transition barrier associated with the switching between the O1, O2 and O3 variants using climbing image nudged elastic band (NEB) method^30^. The result of our calculation for the variant switching between the O1 and O2 variants of WTe_2_ monolayer is shown in Fig. 6. We find the transition barrier of variant switching is only 0.22 eV per formula unit. Very similar results are obtained for orientation switching between other variants, as presented in Supplementary Fig. 2. Note that to facilitate these NEB calculations, we impose supercell strains on the O2 or O3 variants such that they have the same supercell dimensions of the O1 variants. The strain energy results in the slightly higher energy of the O2 or O3 variant that would otherwise be energetically degenerate with the O1 variant. In Supplementary Fig. 3, we have also computed the transition barrier and the pathway between stress-free O1 and O2 variants using generalized solid-state NEB method^31^, which allows both the atomic and supercell degrees of freedom to relax along the transition pathway. The results of the generalized solid-state NEB calculation are very close to those obtained using a fixed-supercell approach, with the calculated energy barrier of variant switching equals to 0.19 eV per formula unit. We note that, while the transition pathway illustrated in Fig. 6 may not be the only possible one, if other pathways exist, the transformation barrier of variant switching can only be smaller or equal than the values we have obtained.

In Supplementary Fig. 4, we have also computed the transition barriers for other MX_2_ monolayers, and the barriers obtained are even lower than 1T′–WTe_2_ monolayers. The transition barriers of variant switching are significantly lower than the barriers of phase transition between the 2H and 1T′ phase^19^, which we computed to be 0.8 eV per formula unit for 1T′–WTe_2_ monolayers at the equilibrium lattice constants of the 2H phase. The much smaller transition barriers associated with the variant switching within the 1T′ phase as compared with the 1T′ to 2H phase transition has an intuitive geometric explanation. Variant switching between the orientation variants of 1T′ phase only involves the distortion of M–X octahedral coordination, while the 1T' to 2H phase transition requires the complete change of M–X coordination pattern from octahedral to trigonal prismatic.

According to transition-state theory, assuming a characteristic attempt frequency of 10 THz, which is the typical frequency of optical phonons in 1T′–MX_2_ monolayers^22^, a 0.2 eV barrier is associated with a timescale of around 0.2 ns. Although the actual barrier of forming a critical nucleus of new variant may involve multiple formula units, and other factors such as interfaces and pre-existing defects may also affect the transformation kinetics, the much smaller barrier associated with variant switching within the 1T′ phase as compared with 2H to 1T′ phase transition^19^,^20^ suggests that ferroelastic variant switching in 1T′–MX_2_ monolayers is very likely to happen under normal laboratory experimental conditions.

### Ferroelastic domain boundaries

**Figure 7:**
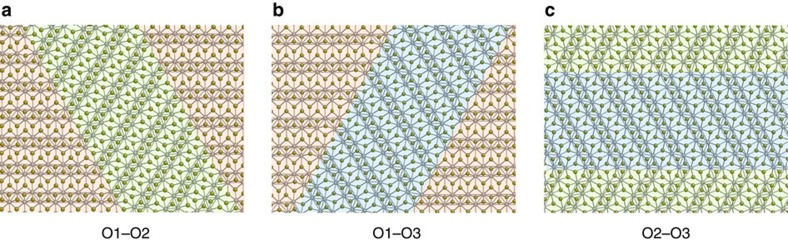
Ferroelastic domain boundaries between different orientation variants. (**a**–**c**) Illustrate the DFT-relaxed atomistic structures of domain boundaries formed between the O1 and O2, O1 and O3, and O2 and O3 variants of 1T′–WTe_2_ monolayers, respectively. To help guide the eyes, domains of different variants are shaded in distinct colours: O1 variant in orange, O2 variant in green and O3 variant in blue.

A direct consequence of strain-induced variant switching in 1T′–MX_2_ monolayers is the formation of domain boundaries between different orientation variants. Strain-induced ferroelastic switching between the O1, O2 and O3 variants can lead to the formation of three possible types of coherent twin boundaries, between O1 and O2, O1 and O3, and between O2 and O3, which we refer to as O1–O2, O1–O3 and O2–O3, respectively. The DFT-relaxed atomistic structures of the three different types of twinning domain boundaries in 1T′–WTe_2_ monolayers under zero external stress are shown in Fig. 7. The three domain boundaries are energetically degenerate, and they are related to each other by 120° rotational symmetry operation. Unlike their three-dimensional (3D) counterparts, where the domain boundaries are 2D, the boundaries formed between the domains of 2D MX_2_ monolayers are quasi-one dimensional (1D) in nature, which may impart them unique properties. We have calculated the domain boundary energies associated with the three types of the 1D domain boundaries and found they have small formation energies. Our DFT calculations give the domain boundary energies of MoS_2_, MoSe_2_, MoTe_2_, WS_2_, WSe_2_ and WTe_2_ monolayers to be 27, 46, 40, 22, 51 and 52 meV· Å^−1^, respectively. In comparison, the formation energy of another type of 1D defects in 3D crystals, dislocations, is in the order of several hundred meV per Angstrom. Such small-domain boundary energies will facilitate the ferroelastic switching between different orientation variants.

## Discussion

The thermodynamic and kinetic analysis above have provided strong evidence that strain-induced ferroelastic switching of orientation variants can occur in 1T′–MX_2_ monolayers, with a few percent of local strain. Our calculations indicate that variant switching can most easily happen when stretching the 1T′–MX_2_ monolayers along the direction of dimerized metal chains. This prediction, if experimentally realized, will render 1T′–MX_2_ monolayers as the first class of 2D ferroelastic materials^32^. Signatures of such ferroelastic switching in experiments include hysteresis in stress–strain curves^27^, and the existence of a force plateau when the externally applied strain is beyond a critical value that corresponds to the onset of variant coexistence. Direct experimental proof of ferroelastic domain switching may be realized by carrying out *in situ* transmission electron microscopy experiments of mechanical deformation of 1T′–MX_2_ monolayers. As the domains of different variants have different crystallographic orientations, the migration of domain walls during strain-induced variant switching can be observed by dark-field transmission electron microscopy, which has been demonstrated for domain imagining in graphene and MoS_2_ monolayers^33^,^34^. Selective area electron diffraction could also reveal the formation of twinning domains, as variant switching results in the rotation of the underlying Bravais lattice of the 1T′ structure, which will manifest in selective area electron diffraction as the rotation of diffraction patterns.

Our prediction of ferroelasticity in the TMD monolayers can be readily tested experimentally in 1T′–WTe_2_ and 1T′–MoTe_2_, for which bulk single crystals have been synthesized on a large scale and exfoliated down to the monolayer or few-layer regime^20^,^21^. Recently, large-area and high-quality MoTe_2_ few layers in 1T′ phase have been grown via chemical vapour deposition^23^,^24^. Local and controlled phase transformation of MoTe_2_ from the 2H to 1T′ phase can also be realized using laser ablation^35^. In principle, ferroelastic domain switching can be observed not only in monolayers but also in few-layer samples, since dimerized metal chains within different layers of the 1T′ phase orient along the same direction in naturally grown crystals^36^.

For other group VI MX_2_ that include MoS_2_, MoSe_2_, WS_2_ and WSe_2_, as the 2H phase is energetically more stable than the 1T′ phase under normal conditions, the 1T′ phase can be realized using a phase engineering approach^37^,^38^. The 1T or 1T′ phase of these materials are now actively being explored for applications in energy and electronics^38^. Monolayers of WS_2_, MoS_2_ and MoSe_2_ in 1T′ phase have been obtained via liquid-phase exfoliation of the bulk crystals intercalated by alkaline metals^18^,^25^,^39^. The transformation from the 2H phase to 1T′ phase by alkaline metal intercalation is attributed to charge transfer from the intercalated alkali atoms to the TMDs^37^. We have performed DFT calculations to study the effect of lithium atom adsorption on the relative energetics of 2H–MX_2_ and 1T′–MX_2_ monolayers, and it is indeed shown that, with increased amount of adsorbed lithium, the 1T′ phase becomes energetically more favourable than the 2H phase for all the MX_2_ monolayers, as illustrated in Supplementary Fig. 5. It has also been proposed that the substitutional doping of MX_2_ with elements having more valence electrons (for example, Re) than the transition metal ions can be another effective way to stabilize the 1T′ phase^40^.

From an application perspective, ferroelastic behaviours have close connection to the shape memory effect, which has been exploited to make actuators in a wide range of industries. In 3D, shape memory alloys (SMAs) is a well known and technologically important class of ferroelastic materials. SMAs can undergo a diffusionless martensitic phase transformation below a critical temperature, from the high-temperature austenite to the low-temperature martensite phase^41^. The martensite phase of SMAs is ferroelastic: it has several equivalent orientations or variants, that can be switched from one to another by an appropriate uniaxial or shear stress. The martensite phase in SMAs can undergo large inelastic deformation through stress-induced migration of twin boundaries between different variants. On heating the deformed crystal above the martensitic phase transformation temperature, the martensite phase can revert to the austenite phase and recover its original shape before deformation. For the ferroelastic 1T′–MX_2_ monolayers, if the 1T′ phase can be reversibly transformed to the 1T or 2H phase under external stimuli (which do not have to be thermal but could be other fields), then MX_2_ monolayers could be 2D shape memory materials, with operating principles similar to SMAs. As such, 1T′–MX_2_ monolayers can be used to make ultrathin actuators for applications in nanoscale-integrated electromechanical systems.

In closing, we would like to make a few additional comments. First, since the 1T′–MX_2_ monolayers were predicted to be quantum spin Hall insulators^22^, topological effect plays an important role in determining their electronic properties. The twinning domain boundaries formed through ferroelastic switching in the 1T′ phase, which are 1D defects in 2D quantum materials, may possess exotic physics and provide a rich playground for domain boundary engineering^42^. Second, the possibility of ferroelastic switching in 2D materials may not be limited to group VI MX_2_ monolayers, considering the rich family of 2D materials^43^. Our initial studies indicate that several other TMD monolayers, including ReS_2_, NbTe_2_ and TaTe_2_, which have low-symmetry distorted crystal structures similar to 1T′^16^, also possess distinct orientation variants and could be ferroelastic as well. Indeed, experimental evidence of local strain-induced orientational switching in ReS_2_ and ReSe_2_ monolayers has recently been reported^44^. Our finding of potential 2D ferroelastic behaviours in monolayer materials could therefore open doors to many exciting discoveries in 2D materials with low-symmetry distorted crystal structures, which may also include ferroelectric, ferromagnetic and multiferroic behaviours in the future.

## Methods

### First-principles calculations

DFT calculations were performed using the Vienna Ab initio Simulation Package with a plane-wave basis set^45^,^46^ and the projector-augmented wave^47^ pseudopotentials. Exchange-correlation effects were treated using the generalized gradient approximation^48^ in the Perdew–Burke–Ernzerhof form^49^. The kinetic energy cutoff for wavefunction expansion was fixed to be 350 eV. The TMD monolayers were modelled in supercells with a vacuum region in the direction perpendicular to the 2D planes of the monolayers (the *z* direction). The length of the supercells along the *z* direction was chosen to be 20 Å. Brillouin zone integration employed a Gamma point centred *m* × *n* × 1 Monkhorst-Pack^50^ **k**-point grid and a Gaussian smearing of 50 meV, where the numbers *m* and *n* were chosen such that the **k**-point sampling spacing is <0.1 Å^−1^ along the supercell reciprocal vectors in the *x*–*y* plane. The energy convergence thresholds for electronic and ionic relaxations were 10^−6^ and 10^−5^ eV, respectively. The maximum residual forces resulted from these convergence criteria are smaller than 5 × 10^−3^ eV Å^−1^. We provide the DFT-relaxed atomistic structures of the O1, O2 and O3 variants of 1T′–MX_2_ monolayers in the POSCAR format of Vienna Ab initio Simulation Package, as listed in Supplementary Tables 4–9.

## Additional information

**How to cite this article:** Li, W. *et al*. Ferroelasticity and domain physics in two-dimensional ransition metal dichalcogenide monolayers. *Nat. Commun.* 7:10843 doi: 10.1038/ncomms10843 (2016).

## Supplementary Material

Supplementary Information Supplementary Figures 1-5, Supplementary Tables 1-9 and Supplementary References.
