# Supplementary material for: Ferroelasticity and domain physics in two-dimensional transition metal dichalcogenide monolayers
Source: Nat Commun. 2016 Feb 24;7:10843. doi: 10.1038/ncomms10843 (PMC4770094; doi:10.1038/ncomms10843)
Supplement: Supplementary Information — Supplementary Figures 1-5, Supplementary Tables 1-9 and Supplementary References. [file ncomms10843-s1.pdf]

Supplementary Figures

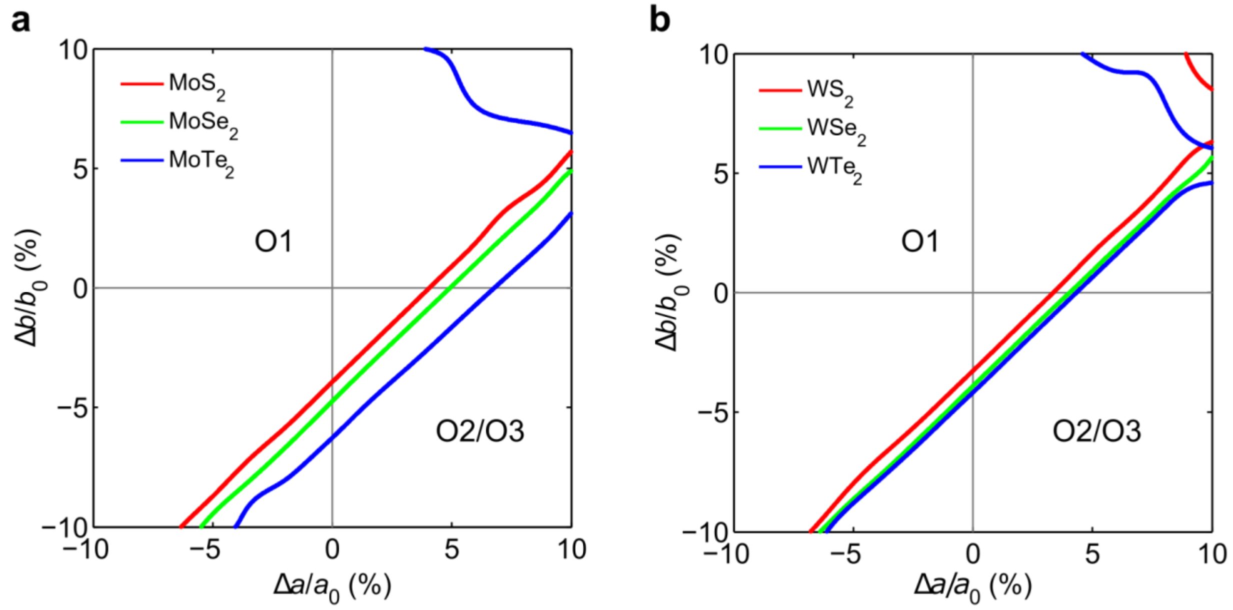

Supplementary Figure 1 | Intersection contours of the potential energy surfaces between the O1 and O2/O3 variants of 1T'−MX_2 monolayers. (a) and (b) show the calculation results for MoX_2 and WX_2 respectively, where X represents S, Se or Te. The lattice constants *a* and *b* of the rectangular 2D unit cell of 1T'−O1 (Figure 1 in main text) are represented as percent engineering strain with respect to the equilibrium lattice constants a_0 and b_0. The O2/O3 variants are forced to adopt the supercell of the O1 variant and their DFT-relaxed energies are compared with the O1 variant. The regions of lower-energy phase are labeled on the plots. Because the 1T' phase is the metastable phase for MX_2 at ambient conditions except for WTe_2, the energies of 1T' phase are in general higher than those of the corresponding 2H phase. Hence, unlike the Figure 3 in main text, the intersection boundaries between 1T' and 2H either lay beyond the range of strain represented in the above plots (for MoS_2, $MoSe_2$, WS_2 and WSe_2), or not shown for consistency ($MoTe_2$).

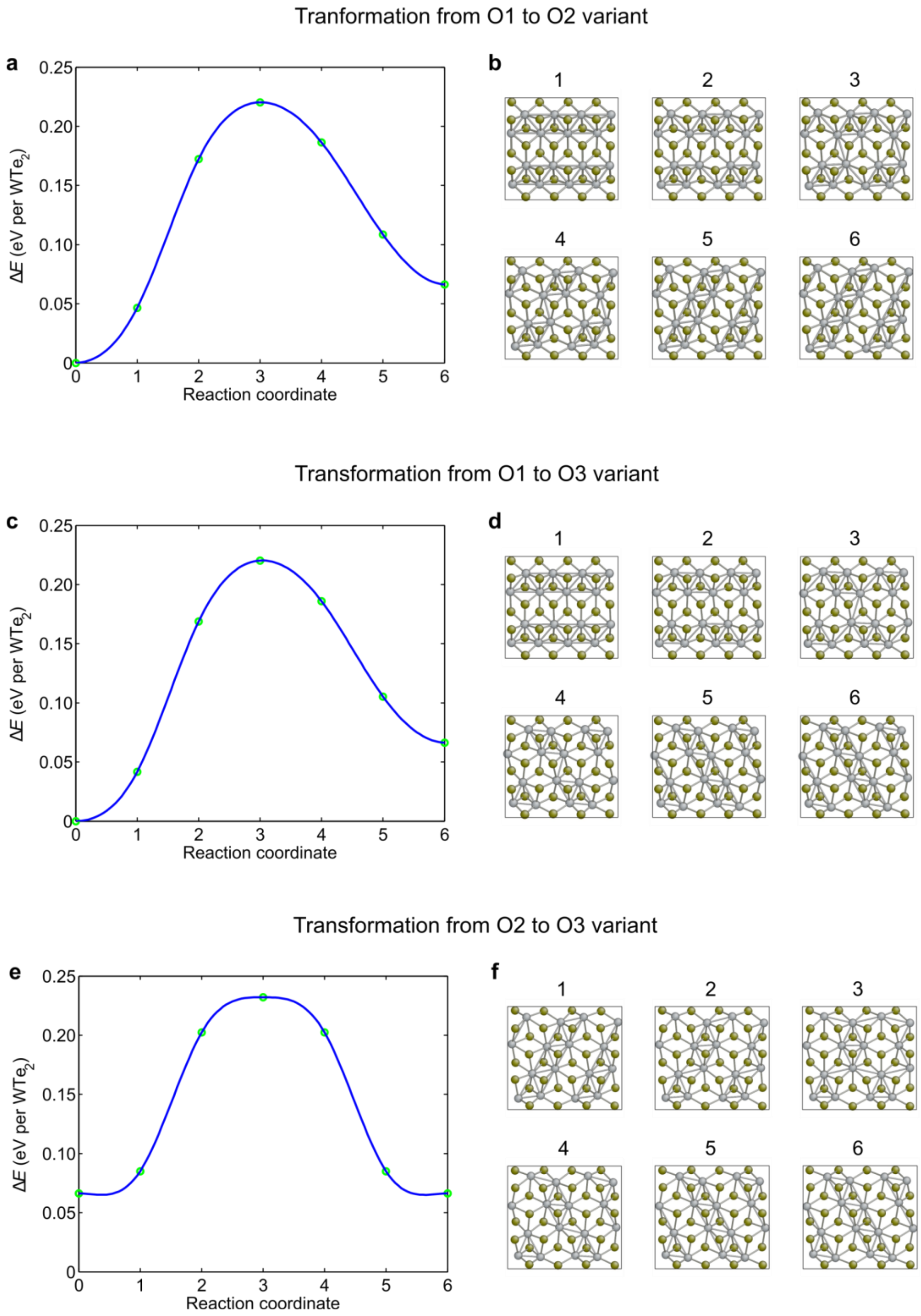

Supplementary Figure 2 | NEB calculation of the transformation barriers and pathways between different orientation variants. (a, b) Variant switching from O1 to O2; (c, d) O1 to O3; (e, f) O2 to O3. In these calculations, the O1 variant is in ground state, while the O2 and O3 are strained to adopt the same supercell vectors as those of the O1 variant. The reference energy is the ground state energy of the three variants, which are the same in zero-strain configurations.

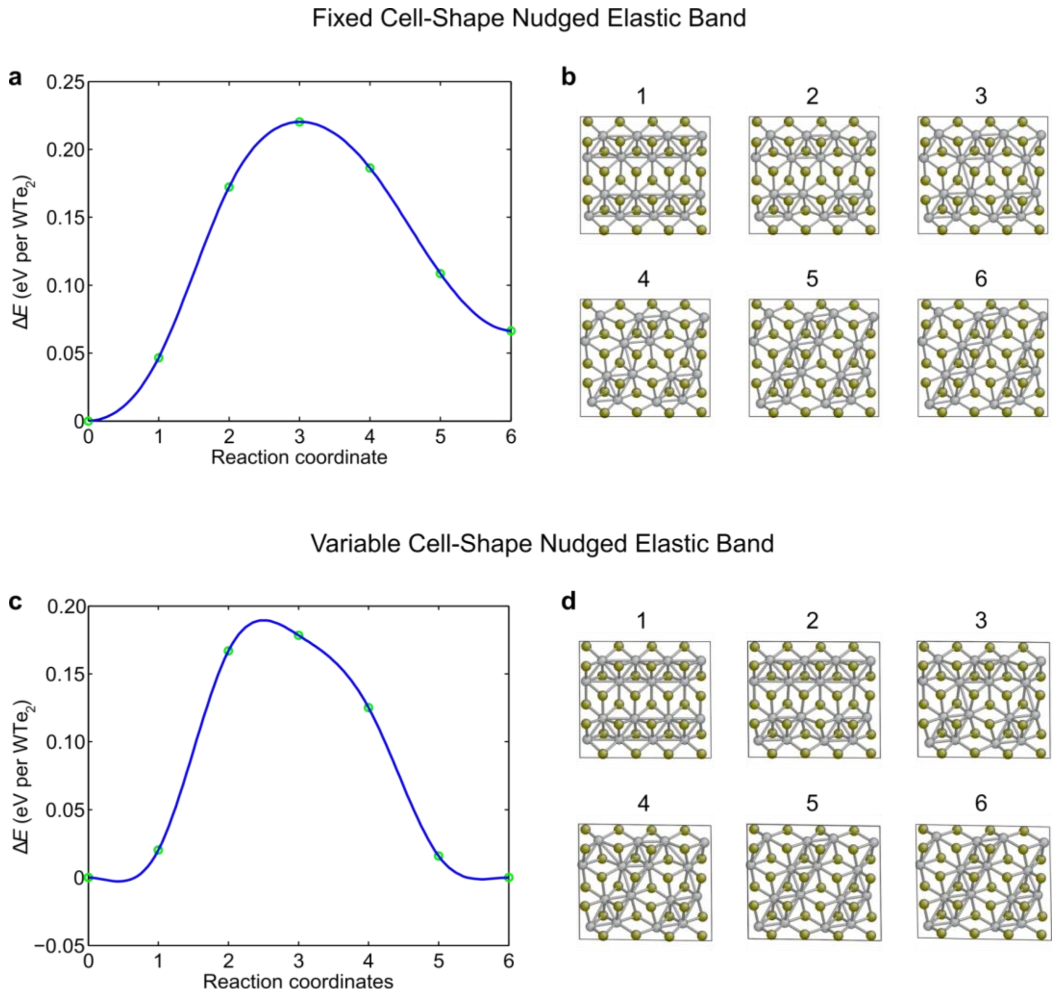

Supplementary Figure 3 | Comparison between fixed and variable cell-shape NEB calculations. The transformation barriers and pathways associated with the variant switching from the O1 to (strained) O2 variant are calculated for 1T'-WTe_2 monolayers. In (a) and (b), the calculations were carried out using climbing image NEB[1] where the initial configuration of the reaction pathway is the ground state of the O1 variant, and the final configuration is the O2 variant strained to adopt the same supercell vectors of the O1 variant. In (c) and (d), both the initial and final configurations are in their strain-free ground state, and therefore have equal system energy but slightly different supercell vectors. Calculations for (c) and (d) were performed using generalized solid-state NEB (G-SSNEB)[2], which allows both the atomic and unit cell degrees of freedom to relax along the transformation pathway.

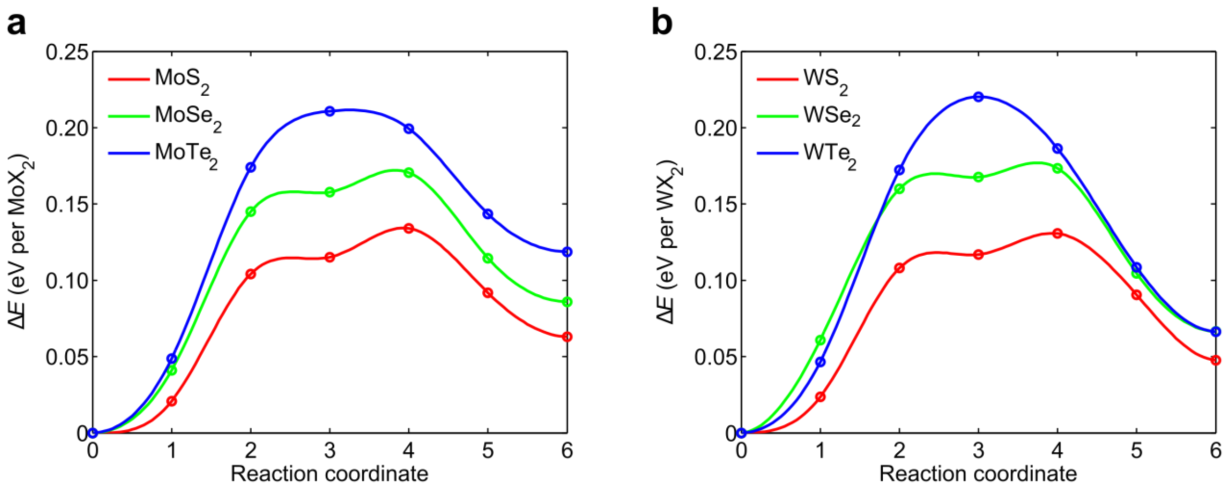

Supplementary Figure 4 | Transformation barriers of MX_2 monolayers from 1T'−O1 to 1T'−O2 as calculated by nudged elastic band (NEB) method. (a) and (b) show the results for MoX_2 and WX_2 monolayers respectively. The supercell for NEB calculation was fixed at the equilibrium (zero-stress) supercell of the O1 variant. The end state of the transformation is O2 variant adopting the supercell geometry of O1 variant, with DFT-relaxed atomic coordinates.

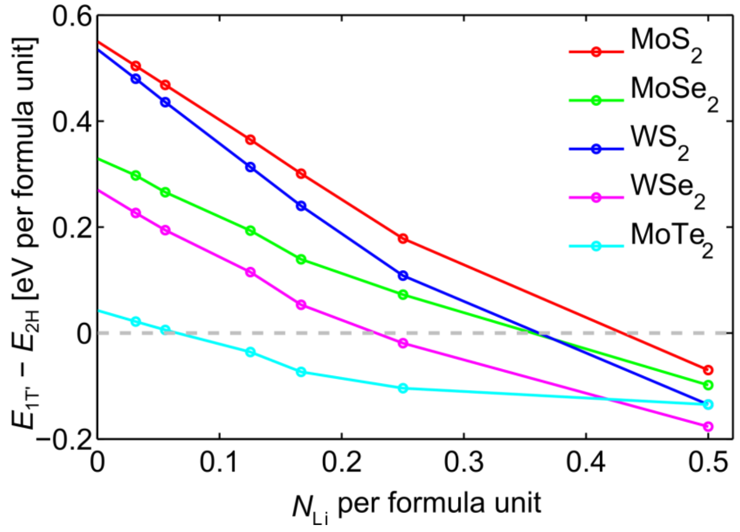

Supplementary Figure 5 | Stabilization of 1T'-MX_2 monolayers *via* lithium atom adsorption. The favorable binding site of the lithium atoms on the surface of the monolayers and the energies of the 2H and 1T' phase are determined *via* DFT calculations. The horizontal axis represents the number for adsorbed lithium atoms per formula unit of MX_2, while the vertical axis denotes the calculated energy difference between the 1T' and 2H phase.

Supplementary Tables

	1T	1T’		
		O1 variant	O2 variant	O3 variant
MoS_2	$\begin{pmatrix} 6.36 & 0.0 \\ 0.0 & 11.01 \end{pmatrix}$	$\begin{pmatrix} 6.34 & 0.0 \\ 0.0 & 11.44 \end{pmatrix}$	$\begin{pmatrix} 6.54 & -0.19 \\ -0.11 & 11.10 \end{pmatrix}$	$\begin{pmatrix} 6.54 & 0.19 \\ 0.11 & 11.10 \end{pmatrix}$
$MoSe_2$	$\begin{pmatrix} 6.55 & 0.0 \\ 0.0 & 11.35 \end{pmatrix}$	$\begin{pmatrix} 6.56 & 0.0 \\ 0.0 & 11.50 \end{pmatrix}$	$\begin{pmatrix} 6.80 & -0.24 \\ -0.14 & 11.50 \end{pmatrix}$	$\begin{pmatrix} 6.80 & 0.24 \\ 0.14 & 11.50 \end{pmatrix}$
$MoTe_2$	$\begin{pmatrix} 6.98 & 0.0 \\ 0.0 & 12.10 \end{pmatrix}$	$\begin{pmatrix} 6.89 & 0.0 \\ 0.0 & 12.73 \end{pmatrix}$	$\begin{pmatrix} 7.24 & -0.35 \\ -0.20 & 12.14 \end{pmatrix}$	$\begin{pmatrix} 7.24 & 0.35 \\ 0.20 & 12.14 \end{pmatrix}$
WS_2	$\begin{pmatrix} 6.39 & 0.0 \\ 0.0 & 11.08 \end{pmatrix}$	$\begin{pmatrix} 6.38 & 0.0 \\ 0.0 & 11.42 \end{pmatrix}$	$\begin{pmatrix} 6.54 & -0.16 \\ -0.09 & 11.14 \end{pmatrix}$	$\begin{pmatrix} 6.54 & 0.16 \\ 0.09 & 11.14 \end{pmatrix}$
WSe_2	$\begin{pmatrix} 6.56 & 0.0 \\ 0.0 & 11.37 \end{pmatrix}$	$\begin{pmatrix} 6.59 & 0.0 \\ 0.0 & 11.88 \end{pmatrix}$	$\begin{pmatrix} 6.79 & -0.20 \\ -0.12 & 11.54 \end{pmatrix}$	$\begin{pmatrix} 6.79 & 0.20 \\ 0.11 & 11.54 \end{pmatrix}$
WTe_2	$\begin{pmatrix} 7.01 & 0.0 \\ 0.0 & 12.14 \end{pmatrix}$	$\begin{pmatrix} 6.98 & 0.0 \\ 0.0 & 12.61 \end{pmatrix}$	$\begin{pmatrix} 7.21 & -0.23 \\ -0.13 & 12.22 \end{pmatrix}$	$\begin{pmatrix} 7.21 & 0.23 \\ 0.13 & 12.23 \end{pmatrix}$

Supplementary Table 1 | Supercell matrices of MX_2 monolayers in 1T and 1T’ phase. The DFT-calculated 2D supercell matrices $\mathbf{H}$ of the $2 \times 2\sqrt{3}$ supercells of 1T−MX_2 monolayers and the corresponding supercells in the 1T’ phase are listed. The three 1T’ variants, O1, O2 and O3, derive from the three symmetry-equivalent directions of structural distortion in the corresponding 1T phase. The supercell matrices have the form $\mathbf{H} = [\boldsymbol{h}_1, \boldsymbol{h}_2]$, where the column vectors $\boldsymbol{h}_1$and $\boldsymbol{h}_2$ are the two basis vectors of the supercells in the 2D planes of MX_2 (see main text for details). The unit of length is Angstrom.

	$\boldsymbol{\eta}_1$ (1T to 1T'-O1)	$\boldsymbol{\eta}_2$ (1T to 1T'-O2)	$\boldsymbol{\eta}_3$ (1T to 1T'-O3)
MoS_2	$\begin{pmatrix} -0.002 & 0.0 \\ 0.0 & 0.039 \end{pmatrix}$	$\begin{pmatrix} 0.030 & -0.018 \\ -0.018 & 0.009 \end{pmatrix}$	$\begin{pmatrix} 0.030 & 0.018 \\ 0.018 & 0.009 \end{pmatrix}$
$MoSe_2$	$\begin{pmatrix} 0.001 & 0.0 \\ 0.0 & 0.051 \end{pmatrix}$	$\begin{pmatrix} 0.039 & -0.022 \\ -0.022 & 0.013 \end{pmatrix}$	$\begin{pmatrix} 0.039 & 0.022 \\ 0.022 & 0.013 \end{pmatrix}$
$MoTe_2$	$\begin{pmatrix} -0.013 & 0.0 \\ 0.0 & 0.054 \end{pmatrix}$	$\begin{pmatrix} 0.038 & -0.030 \\ -0.030 & 0.004 \end{pmatrix}$	$\begin{pmatrix} 0.038 & 0.029 \\ 0.029 & 0.004 \end{pmatrix}$
WS_2	$\begin{pmatrix} -0.002 & 0.0 \\ 0.0 & 0.031 \end{pmatrix}$	$\begin{pmatrix} 0.023 & -0.015 \\ -0.015 & 0.006 \end{pmatrix}$	$\begin{pmatrix} 0.023 & 0.015 \\ 0.015 & 0.006 \end{pmatrix}$
WSe_2	$\begin{pmatrix} 0.004 & 0.0 \\ 0.0 & 0.046 \end{pmatrix}$	$\begin{pmatrix} 0.035 & -0.018 \\ -0.018 & 0.015 \end{pmatrix}$	$\begin{pmatrix} 0.035 & 0.018 \\ 0.018 & 0.015 \end{pmatrix}$
WTe_2	$\begin{pmatrix} -0.005 & 0.0 \\ 0.0 & 0.039 \end{pmatrix}$	$\begin{pmatrix} 0.029 & -0.019 \\ -0.019 & 0.006 \end{pmatrix}$	$\begin{pmatrix} 0.029 & 0.019 \\ 0.019 & 0.007 \end{pmatrix}$

Supplementary Table 2 | Spontaneous strain of 1T to 1T' phase transition in MX_2 monolayers. The spontaneous strain matrices $\boldsymbol{\eta}$ associated with the structural transition from the 1T phase to the O1, O2 and O3 variants of 1T' phase are listed for different MX_2 monolayers.

	$\boldsymbol{\varepsilon}_1^2$ (O1 to O2)	$\boldsymbol{\varepsilon}_1^3$ (O1 to O3)
MoS_2	$\begin{pmatrix} 0.031 & -0.017 \\ -0.017 & -0.029 \end{pmatrix}$	$\begin{pmatrix} 0.031 & 0.017 \\ 0.017 & -0.029 \end{pmatrix}$
$MoSe_2$	$\begin{pmatrix} 0.038 & -0.021 \\ -0.021 & -0.034 \end{pmatrix}$	$\begin{pmatrix} 0.038 & 0.021 \\ 0.021 & -0.034 \end{pmatrix}$
$MoTe_2$	$\begin{pmatrix} 0.053 & -0.029 \\ -0.029 & -0.045 \end{pmatrix}$	$\begin{pmatrix} 0.053 & 0.028 \\ 0.028 & -0.045 \end{pmatrix}$
WS_2	$\begin{pmatrix} 0.026 & -0.015 \\ -0.015 & -0.024 \end{pmatrix}$	$\begin{pmatrix} 0.026 & 0.015 \\ 0.015 & -0.024 \end{pmatrix}$
WSe_2	$\begin{pmatrix} 0.031 & -0.017 \\ -0.017 & -0.028 \end{pmatrix}$	$\begin{pmatrix} 0.031 & 0.017 \\ 0.017 & -0.028 \end{pmatrix}$
WTe_2	$\begin{pmatrix} 0.034 & -0.019 \\ -0.019 & -0.030 \end{pmatrix}$	$\begin{pmatrix} 0.033 & 0.019 \\ 0.019 & -0.030 \end{pmatrix}$

Supplementary Table 3 | Transformation strain between different orientation variants. The Transformation strain matrices $\boldsymbol{\varepsilon}$ associated with the variant switching from O1 to O2 variant and O1 to O3 variant are listed for different 1T'-MX_2 monolayers.

MoS2 1T’−O1	MoS2 1T’−O2	MoS2 1T’−O3
1.0	1.0	1.0
6.344802 0.000000 0.000000	6.539611 -0.112305 0.000000	6.539654 0.111960 0.000000
0.000000 11.436521 0.000000	-0.193172 11.103473 0.000000	0.193944 11.103862 0.000000
0.000000 0.000000 20.000000	0.000000 0.000000 20.000000	0.000000 0.000000 20.000000
Mo S	Mo S	Mo S
8 16	8 16	8 16
Direct	Direct	Direct
0.000000 0.017713 0.496032	0.049431 0.983463 0.496093	0.023511 0.007926 0.504010
0.250000 0.215620 0.504002	0.299598 0.233431 0.496024	0.195287 0.231687 0.495999
0.000000 0.517713 0.496032	0.971210 0.509578 0.504007	0.945302 0.481697 0.496068
0.250000 0.715620 0.504002	0.221371 0.759588 0.503956	0.273435 0.757876 0.503993
0.500000 0.017713 0.496032	0.471189 0.009566 0.504052	0.445294 0.981696 0.496063
0.750000 0.215620 0.504002	0.721346 0.259595 0.503979	0.773428 0.257880 0.503966
0.500000 0.517713 0.496032	0.549457 0.483470 0.496054	0.523512 0.507924 0.504021
0.750000 0.715620 0.504002	0.799620 0.733438 0.495996	0.695298 0.731683 0.496027
0.000000 0.327484 0.433522	0.014228 0.328636 0.433555	0.983298 0.327798 0.413117
0.250000 0.075793 0.413122	0.261399 0.079455 0.413154	0.233319 0.077705 0.413119
0.000000 0.157540 0.586920	0.006806 0.164481 0.566504	0.985397 0.161858 0.586892
0.250000 0.405850 0.566519	0.256550 0.414426 0.566476	0.238157 0.412668 0.566464
0.000000 0.827484 0.433522	0.011612 0.829576 0.413118	0.980737 0.826874 0.433578
0.250000 0.575793 0.413122	0.264107 0.578594 0.433539	0.230634 0.576848 0.433583
0.000000 0.657540 0.586920	0.009471 0.663581 0.586878	0.987992 0.662718 0.566491
0.250000 0.905850 0.566519	0.259210 0.913478 0.586936	0.235437 0.911801 0.586920
0.500000 0.327484 0.433522	0.511602 0.329514 0.413120	0.480738 0.326903 0.433557
0.750000 0.075793 0.413122	0.764100 0.078580 0.433585	0.730634 0.076834 0.433566
0.500000 0.157540 0.586920	0.509467 0.163598 0.586912	0.487994 0.162734 0.566464
0.750000 0.405850 0.566519	0.759214 0.413541 0.586911	0.735445 0.411766 0.586915
0.500000 0.827484 0.433522	0.514245 0.828691 0.433547	0.483294 0.827763 0.413136
0.750000 0.575793 0.413122	0.761401 0.579471 0.413112	0.733314 0.577725 0.413135
0.500000 0.657540 0.586920	0.506828 0.664474 0.566462	0.485390 0.661841 0.586917
0.750000 0.905850 0.566519	0.756540 0.914374 0.566503	0.738153 0.912695 0.566469

Supplementary Table 4 | Relaxed atomic structures of the O1, O2 and O3 orientation variants of 1T’−MoS_2 in the VASP/POSCAR format.

MoSe2 1T’−O1	MoSe2 1T’−O2	MoSe2 1T’−O3
1.0	1.0	1.0
6.557810 0.000000 0.000000	6.800475 -0.141847 0.000000	6.801027 0.140481 0.000000
0.000000 11.918348 0.000000	-0.244194 11.498713 0.000000	0.243343 11.499961 0.000000
0.000000 0.000000 20.000000	0.000000 0.000000 20.000000	0.000000 0.000000 20.000000
Mo Se	Mo Se	Mo Se
8 16	8 16	8 16
Direct	Direct	Direct
0.000000 0.021570 0.495772	0.055344 0.981582 0.495707	0.029083 0.009805 0.504309
0.250000 0.211774 0.504285	0.305226 0.231464 0.495682	0.189655 0.229692 0.495698
0.000000 0.521570 0.495772	0.965748 0.511478 0.504303	0.939341 0.479781 0.495698
0.250000 0.711774 0.504285	0.215467 0.761501 0.504361	0.279369 0.759748 0.504366
0.500000 0.021570 0.495772	0.465724 0.011463 0.504320	0.439336 0.979783 0.495677
0.750000 0.211774 0.504285	0.715465 0.261502 0.504360	0.779357 0.259753 0.504353
0.500000 0.521570 0.495772	0.555390 0.481602 0.495680	0.529067 0.509789 0.504331
0.750000 0.711774 0.504285	0.805232 0.731460 0.495678	0.689636 0.729718 0.495725
0.000000 0.329097 0.429935	0.016650 0.327661 0.430072	0.983378 0.327666 0.406186
0.250000 0.075919 0.406219	0.262279 0.079054 0.406169	0.232385 0.077505 0.406134
0.000000 0.157465 0.593817	0.004469 0.165241 0.570185	0.986488 0.162120 0.593854
0.250000 0.404175 0.570090	0.254507 0.415473 0.569946	0.240443 0.413665 0.569874
0.000000 0.829097 0.429935	0.011687 0.829420 0.406191	0.978302 0.825884 0.430152
0.250000 0.575919 0.406219	0.266993 0.578086 0.429971	0.227977 0.576244 0.430012
0.000000 0.657465 0.593817	0.008187 0.663684 0.593832	0.990547 0.663384 0.570083
0.250000 0.904175 0.570090	0.258438 0.913663 0.593839	0.235574 0.911965 0.593837
0.500000 0.329097 0.429935	0.511692 0.329401 0.406187	0.478303 0.325902 0.430141
0.750000 0.075919 0.406219	0.766991 0.078072 0.429986	0.727989 0.076242 0.430000
0.500000 0.157465 0.593817	0.508188 0.163690 0.593838	0.490543 0.163391 0.570068
0.750000 0.404175 0.570090	0.758441 0.413681 0.593823	0.735556 0.411929 0.593855
0.500000 0.829097 0.429935	0.516645 0.827670 0.430073	0.483398 0.827650 0.406192
0.750000 0.575919 0.406219	0.762335 0.579092 0.406138	0.732320 0.577494 0.406176
0.500000 0.657465 0.593817	0.504457 0.665227 0.570175	0.486498 0.662104 0.593873
0.750000 0.904175 0.570090	0.754445 0.915430 0.569957	0.740453 0.913687 0.569876

Supplementary Table 5 | Relaxed atomic structures of the O1, O2 and O3 orientation variants of 1T’−$MoSe_2$ monolayers in the VASP/POSCAR format.

MoTe2 1T’−O1	MoTe2 1T’−O2	MoTe2 1T’−O3
1.0	1.0	1.0
6.890280 0.000000 0.000000	7.240200 -0.203063 0.000000	7.240880 0.199458 0.000000
0.000000 12.734937 0.000000	-0.349924 12.140670 0.000000	0.345502 12.144272 0.000000
0.000000 0.000000 20.000000	0.000000 0.000000 20.000000	0.000000 0.000000 20.000000
Mo Te	Mo Te	Mo Te
8 16	8 16	8 16
Direct	Direct	Direct
0.000000 0.025986 0.495485	0.061897 0.979331 0.495308	0.036037 0.011940 0.504752
0.250000 0.207341 0.504551	0.311910 0.229341 0.495306	0.182808 0.227551 0.495285
0.000000 0.525986 0.495485	0.958776 0.513641 0.504742	0.932806 0.477552 0.495292
0.250000 0.707341 0.504551	0.208774 0.763624 0.504746	0.286028 0.761948 0.504764
0.500000 0.025986 0.495485	0.458768 0.013619 0.504772	0.432794 0.977557 0.495291
0.750000 0.207341 0.504551	0.708769 0.263626 0.504758	0.786052 0.261940 0.504747
0.500000 0.525986 0.495485	0.561925 0.479328 0.495267	0.536036 0.511949 0.504761
0.750000 0.707341 0.504551	0.811907 0.729332 0.495287	0.682789 0.727555 0.495300
0.000000 0.331742 0.425799	0.021250 0.326431 0.426039	0.979744 0.326765 0.396711
0.250000 0.076934 0.396727	0.265292 0.078370 0.396745	0.229732 0.076761 0.396713
0.000000 0.156418 0.603329	0.999792 0.166743 0.573925	0.988674 0.162813 0.603382
0.250000 0.401579 0.574228	0.249814 0.416758 0.573913	0.244891 0.414852 0.573843
0.000000 0.831742 0.425799	0.015275 0.828395 0.396715	0.974122 0.824787 0.426121
0.250000 0.576934 0.396727	0.271253 0.576433 0.426003	0.224140 0.574785 0.426121
0.000000 0.656418 0.603329	0.005491 0.664637 0.603405	0.994881 0.664858 0.573853
0.250000 0.901579 0.574228	0.255493 0.914630 0.603415	0.238654 0.912821 0.603393
0.500000 0.331742 0.425799	0.515277 0.328368 0.396716	0.474148 0.324788 0.426111
0.750000 0.076934 0.396727	0.771244 0.076434 0.426045	0.724126 0.074789 0.426112
0.500000 0.156418 0.603329	0.505505 0.164639 0.603417	0.494892 0.164866 0.573840
0.750000 0.401579 0.574228	0.755498 0.414651 0.603398	0.738671 0.412803 0.603387
0.500000 0.831742 0.425799	0.521240 0.826445 0.426033	0.479729 0.826756 0.396723
0.750000 0.576934 0.396727	0.765279 0.578377 0.396704	0.729737 0.576765 0.396723
0.500000 0.656418 0.603329	0.499782 0.666720 0.573887	0.488653 0.662812 0.603398
0.750000 0.901579 0.574228	0.749789 0.916725 0.573925	0.744855 0.914884 0.573848

Supplementary Table 6 | Relaxed atomic structures of the O1, O2 and O3 orientation variants of 1T’−$MoTe_2$ monolayers in the VASP/POSCAR format.

WS2 1T’−O1	WS2 1T’−O2	WS2 1T’−O3
1.0	1.0	1.0
6.376993 0.000000 0.000000	6.538735 -0.094673 0.000000	6.538940 0.094283 0.000000
0.000000 11.415126 0.000000	-0.162651 11.137554 0.000000	0.163309 11.137920 0.000000
0.000000 0.000000 20.000000	0.000000 0.000000 20.000000	0.000000 0.000000 20.000000
W S	W S	W S
8 16	8 16	8 16
Direct	Direct	Direct
0.000000 0.017492 0.496394	0.049184 0.983562 0.496492	0.023220 0.007717 0.503529
0.250000 0.215836 0.503646	0.299238 0.233568 0.496505	0.195516 0.231786 0.496478
0.000000 0.517492 0.496394	0.971552 0.509468 0.503525	0.945516 0.481800 0.496487
0.250000 0.715836 0.503646	0.221633 0.759485 0.503555	0.273236 0.757749 0.503585
0.500000 0.017492 0.496394	0.471535 0.009457 0.503558	0.445508 0.981801 0.496482
0.750000 0.215836 0.503646	0.721629 0.259483 0.503570	0.773235 0.257752 0.503558
0.500000 0.517492 0.496394	0.549211 0.483566 0.496461	0.523220 0.507711 0.503542
0.750000 0.715836 0.503646	0.799246 0.733576 0.496486	0.695518 0.731779 0.496506
0.000000 0.326867 0.433524	0.013363 0.328878 0.433558	0.985419 0.328541 0.412738
0.250000 0.074583 0.412747	0.259246 0.080253 0.412758	0.235515 0.078515 0.412720
0.000000 0.158754 0.587292	0.007643 0.164185 0.566502	0.983320 0.161079 0.587289
0.250000 0.406467 0.566514	0.257458 0.414184 0.566467	0.237296 0.412428 0.566471
0.000000 0.826867 0.433524	0.009423 0.830277 0.412742	0.981540 0.827132 0.433564
0.250000 0.574583 0.412747	0.263247 0.578876 0.433541	0.231479 0.577151 0.433565
0.000000 0.658754 0.587292	0.011590 0.662786 0.587285	0.987172 0.662462 0.566500
0.250000 0.906467 0.566514	0.261420 0.912776 0.587306	0.233281 0.911098 0.587308
0.500000 0.326867 0.433524	0.509422 0.330227 0.412744	0.481542 0.327160 0.433545
0.750000 0.074583 0.412747	0.763243 0.078862 0.433576	0.731479 0.077140 0.433546
0.500000 0.158754 0.587292	0.511600 0.162798 0.587313	0.487176 0.162479 0.566471
0.750000 0.406467 0.566514	0.761423 0.412821 0.587286	0.733299 0.411062 0.587304
0.500000 0.826867 0.433524	0.513360 0.828915 0.433552	0.485406 0.828507 0.412758
0.750000 0.574583 0.412747	0.759250 0.580270 0.412725	0.735503 0.578529 0.412738
0.500000 0.658754 0.587292	0.507642 0.664177 0.566473	0.483311 0.661065 0.587314
0.750000 0.906467 0.566514	0.757444 0.914146 0.566491	0.737294 0.912453 0.566472

Supplementary Table 7 | Relaxed atomic structures of the O1, O2 and O3 orientation variants of 1T’−WS_2 monolayers in the VASP/POSCAR format.

WSe2 1T’−O1	WSe2 1T’−O2	WSe2 1T’−O3
1.0	1.0	1.0
6.592315 0.000000 0.000000	6.791149 -0.115482 0.000000	6.790538 0.114009 0.000000
0.000000 11.879572 0.000000	-0.198560 11.535637 0.000000	0.197449 11.535890 0.000000
0.000000 0.000000 20.000000	0.000000 0.000000 20.000000	0.000000 0.000000 20.000000
W Se	W Se	W Se
8 16	8 16	8 16
Direct	Direct	Direct
0.000000 0.022068 0.495735	0.056244 0.981268 0.495623	0.029895 0.010133 0.504349
0.250000 0.211159 0.504303	0.306087 0.231180 0.495671	0.188798 0.229486 0.495669
0.000000 0.522068 0.495735	0.964692 0.511777 0.504318	0.938506 0.479543 0.495603
0.250000 0.711159 0.504303	0.214707 0.761813 0.504445	0.280288 0.760090 0.504423
0.500000 0.022068 0.495735	0.464690 0.011771 0.504319	0.438514 0.979565 0.495620
0.750000 0.211159 0.504303	0.714705 0.261813 0.504444	0.780299 0.260090 0.504406
0.500000 0.522068 0.495735	0.556238 0.481269 0.495623	0.529886 0.510144 0.504357
0.750000 0.711159 0.504303	0.806087 0.731183 0.495672	0.688793 0.729480 0.495712
0.000000 0.328345 0.429951	0.015189 0.328061 0.430060	0.984869 0.328092 0.405266
0.250000 0.074832 0.405335	0.260342 0.079957 0.405171	0.234020 0.078077 0.405251
0.000000 0.158600 0.594689	0.005482 0.164773 0.570096	0.984764 0.161381 0.594810
0.250000 0.404995 0.570105	0.255758 0.414956 0.569924	0.239110 0.413241 0.569888
0.000000 0.828345 0.429951	0.010347 0.829779 0.405318	0.979554 0.826270 0.430159
0.250000 0.574832 0.405335	0.265326 0.578394 0.430049	0.229136 0.576574 0.430002
0.000000 0.658600 0.594689	0.010415 0.663040 0.594743	0.989525 0.663072 0.570022
0.250000 0.904995 0.570105	0.260415 0.913304 0.594816	0.234039 0.911434 0.594729
0.500000 0.328345 0.429951	0.510344 0.329780 0.405317	0.479574 0.326302 0.430145
0.750000 0.074832 0.405335	0.765327 0.078392 0.430049	0.729135 0.076567 0.429999
0.500000 0.158600 0.594689	0.510418 0.163038 0.594742	0.489537 0.163092 0.570014
0.750000 0.404995 0.570105	0.760412 0.413306 0.594816	0.734051 0.411427 0.594722
0.500000 0.828345 0.429951	0.515190 0.828061 0.430061	0.484922 0.828103 0.405292
0.750000 0.574832 0.405335	0.760345 0.579960 0.405171	0.734002 0.578100 0.405262
0.500000 0.658600 0.594689	0.505483 0.664772 0.570095	0.484633 0.661355 0.594844
0.750000 0.904995 0.570105	0.755756 0.914954 0.569925	0.739151 0.913279 0.569927

Supplementary Table 8 | Relaxed atomic structures of the O1, O2 and O3 orientation variants of 1T’−WSe_2 monolayers in the VASP/POSCAR format.

WTe2 1T’−O1	WTe2 1T’−O2	WTe2 1T’−O3
1.0	1.0	1.0
6.980712 0.000000 0.000000	7.211354 -0.133713 0.000000	7.208065 0.131540 0.000000
0.000000 12.611188 0.000000	-0.230241 12.215912 0.000000	0.227793 12.226579 0.000000
0.000000 0.000000 20.000000	0.000000 0.000000 20.000000	0.000000 0.000000 20.000000
W Te	W Te	W Te
8 16	8 16	8 16
Direct	Direct	Direct
0.000000 0.027739 0.494860	0.064441 0.978375 0.494871	0.038516 0.012757 0.505238
0.250000 0.205591 0.505178	0.314527 0.228405 0.494840	0.180290 0.226697 0.494710
0.000000 0.527739 0.494860	0.956353 0.514640 0.505169	0.930289 0.476691 0.494757
0.250000 0.705591 0.505178	0.206350 0.764638 0.505217	0.288463 0.762856 0.505329
0.500000 0.027739 0.494860	0.456255 0.014579 0.505296	0.430145 0.976757 0.494790
0.750000 0.205591 0.505178	0.706315 0.264622 0.505264	0.788648 0.262735 0.505217
0.500000 0.527739 0.494860	0.564578 0.478416 0.494732	0.538579 0.512819 0.505325
0.750000 0.705591 0.505178	0.814509 0.728357 0.494746	0.680129 0.726743 0.494821
0.000000 0.330360 0.426153	0.019045 0.326752 0.426415	0.981272 0.326979 0.395381
0.250000 0.076512 0.395453	0.263329 0.078553 0.395441	0.231187 0.076893 0.395385
0.000000 0.156810 0.604577	0.001899 0.166408 0.573760	0.987774 0.162390 0.604610
0.250000 0.402987 0.573896	0.251931 0.416448 0.573687	0.242862 0.414192 0.573604
0.000000 0.830360 0.426153	0.013445 0.828618 0.395375	0.975794 0.825534 0.426365
0.250000 0.576512 0.395453	0.268975 0.576892 0.426312	0.225920 0.575643 0.426415
0.000000 0.656810 0.604577	0.007383 0.664428 0.604565	0.992787 0.664215 0.573623
0.250000 0.902987 0.573896	0.257335 0.914386 0.604658	0.237428 0.912498 0.604713
0.500000 0.330360 0.426153	0.513445 0.328473 0.395373	0.475931 0.325649 0.426340
0.750000 0.076512 0.395453	0.768964 0.076751 0.426490	0.725801 0.075558 0.426317
0.500000 0.156810 0.604577	0.507384 0.164455 0.604639	0.492890 0.164272 0.573589
0.750000 0.402987 0.573896	0.757373 0.414511 0.604585	0.737773 0.412298 0.604628
0.500000 0.830360 0.426153	0.518970 0.826856 0.426388	0.481098 0.826984 0.395471
0.750000 0.576512 0.395453	0.763494 0.578500 0.395254	0.731153 0.577009 0.395482
0.500000 0.656810 0.604577	0.501869 0.666280 0.573625	0.487447 0.662461 0.604736
0.750000 0.902987 0.573896	0.751833 0.916254 0.573766	0.742823 0.914269 0.573623

Supplementary Table 9 | Relaxed atomic structures of the O1, O2 and O3 orientation variants of 1T’−WTe_2 monolayers in the VASP/POSCAR format.

Supplementary References

1. Henkelman, G., Uberuaga, B. P. & Jónsson, H. A climbing image nudged elastic band method for finding saddle points and minimum energy paths. *J. Chem. Phys.* **113,** 9901–9904 (2000)

2. Sheppard, D., Xiao, P., Chemelewski, W., Johnson, D. D. & Henkelman, G. A generalized solid-state nudged elastic band method. *J. Chem. Phys.* **136,** 074103 (2012)
